# Supplementary material for: Statistical Copolymers of N-Vinylpyrrolidone and Isobornyl Methacrylate via Free Radical and RAFT Polymerization: Monomer Reactivity Ratios, Thermal Properties, and Kinetics of Thermal Decomposition
Source: Polymers (Basel). 2021 Mar 3;13(5):778. doi: 10.3390/polym13050778 (PMC7961878; doi:10.3390/polym13050778)
Supplement: Supplementary file 1 [file polymers-13-00778-s001.pdf]

polymers

MDPI

Supplementary Materials

Statistical Copolymers of N-Vinylpyrrolidone and Isobornyl Methacrylate via Free Radical and RAFT Polymerization: Monomer Reactivity Ratios, Thermal Properties, and Kinetics of Thermal Decomposition

Olga Kokkorogianni, Philippos Kontoes-Georgoudakis, Maria Athanasopoulou, Nikolaos Polizos and Marinos Pitsikalis *

Industrial Chemistry Laboratory, Department of Chemistry, National and Kapodistrian University of Athens, Panepistimiopolis Zografou, 15771 Athens, Greece; olga.kokkorogianni@gmail.com (O.K.); kontoes10@hotmail.com (P.K.-G.); athanamaria@gmail.com (M.A.); nikospolyz93@yahoo.g (N.P.)

* Correspondence: pitsikalis@chem.uoa.gr; Tel.: +30-210-727-4440

Citation: Kokkorogianni, O.; Kontoes-Georgoudakis, P.; Athanasopoulou, M.; Polizos, N.; Pitsikalis, M. Statistical Copolymers of N-Vinylpyrrolidone and Isobornyl Methacrylate via Free Radical and RAFT Polymerization: Monomer Reactivity Ratios, Thermal Properties, and Kinetics of Thermal Decomposition. *Polymers* **2021**, *13*, 778. https://doi.org/10.3390/polym13050778

Academic Editor: Asterios (Stergios) Pispas

Received: 8 February 2021

Accepted: 26 February 2021

Published: 3 March 2021

Publisher's Note: MDPI stays neutral with regard to jurisdictional claims in published maps and institutional affiliations.

Copyright: © 2021 by the author. Licensee MDPI, Basel, Switzerland. This article is an open access article distributed under the terms and conditions of the Creative Commons Attribution (CC BY) license (http://creativecommons.org/licenses/by/4.0/).

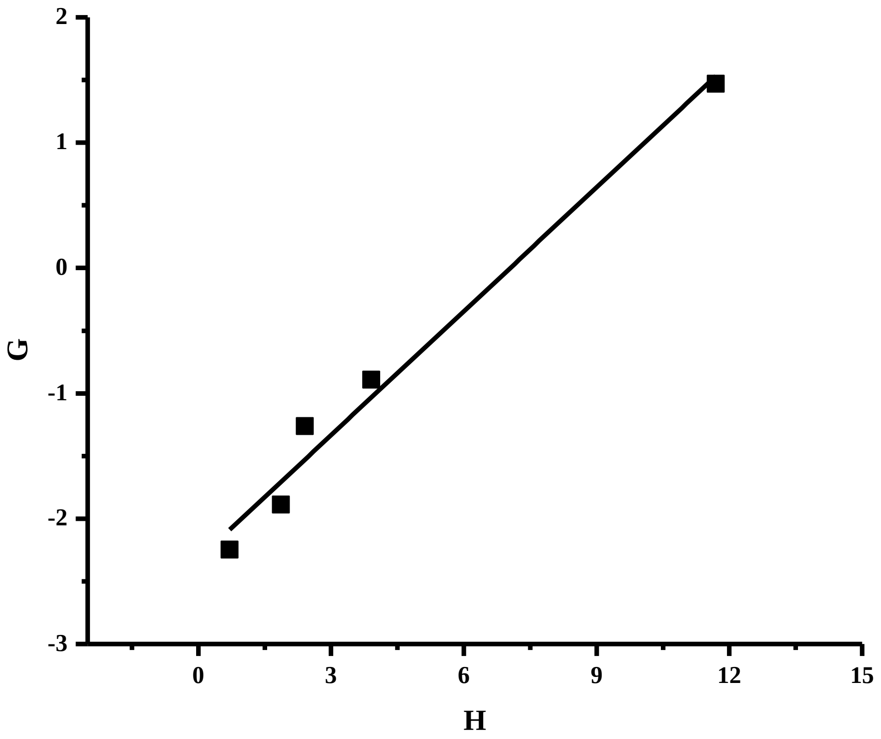

Figure S1. Fineman-Ross plot for the PNVP-stat-PIBMA copolymers synthesized via free radical copolymerization.

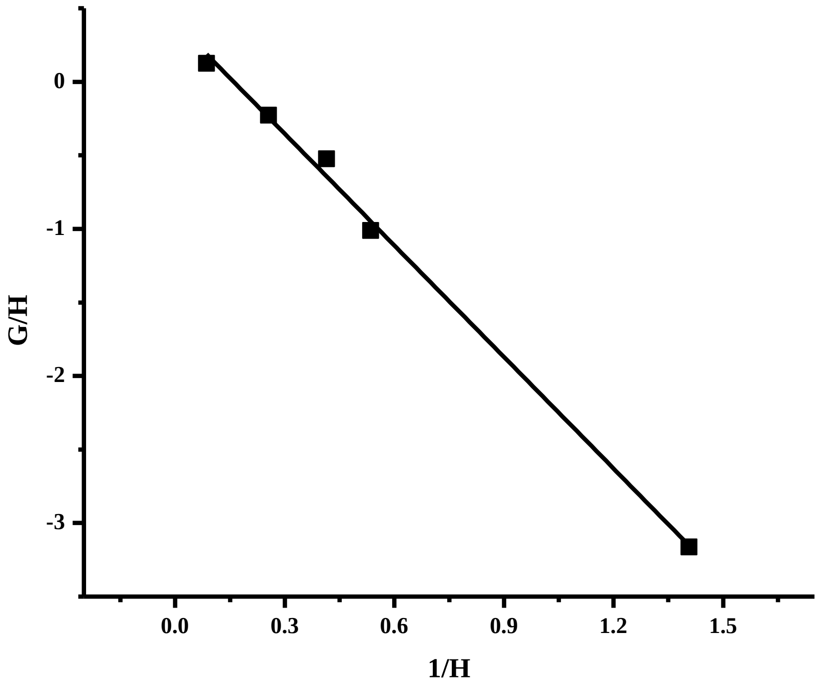

Figure S2. Inverted Fineman-Ross plot for the PNVP-stat-PIBMA copolymers synthesized via free radical copolymerization.

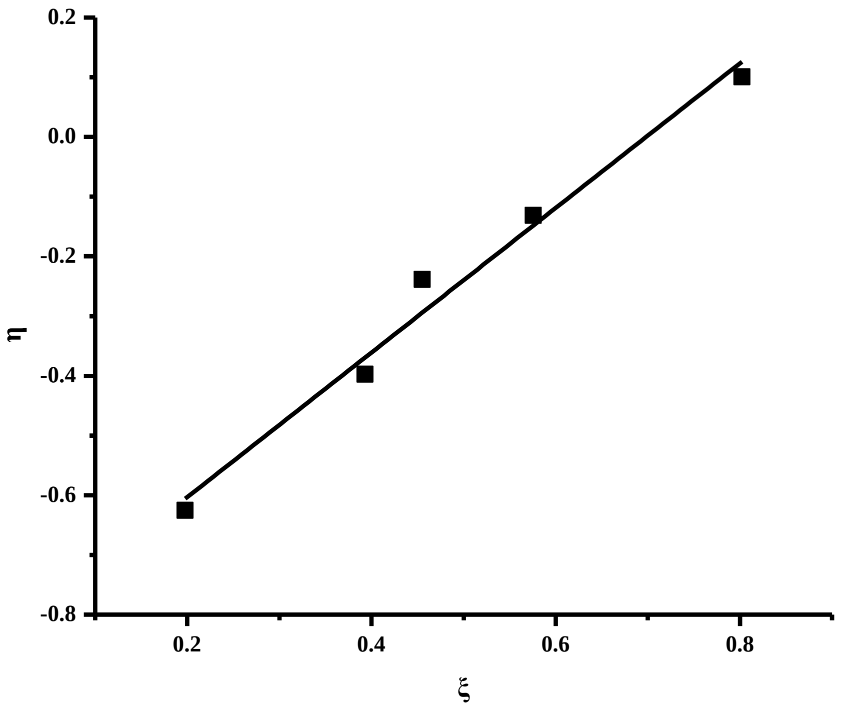

Figure S3. Kelen-Tüdos plot for the PNVP-stat-PIBMA copolymers synthesized via free radical copolymerization.

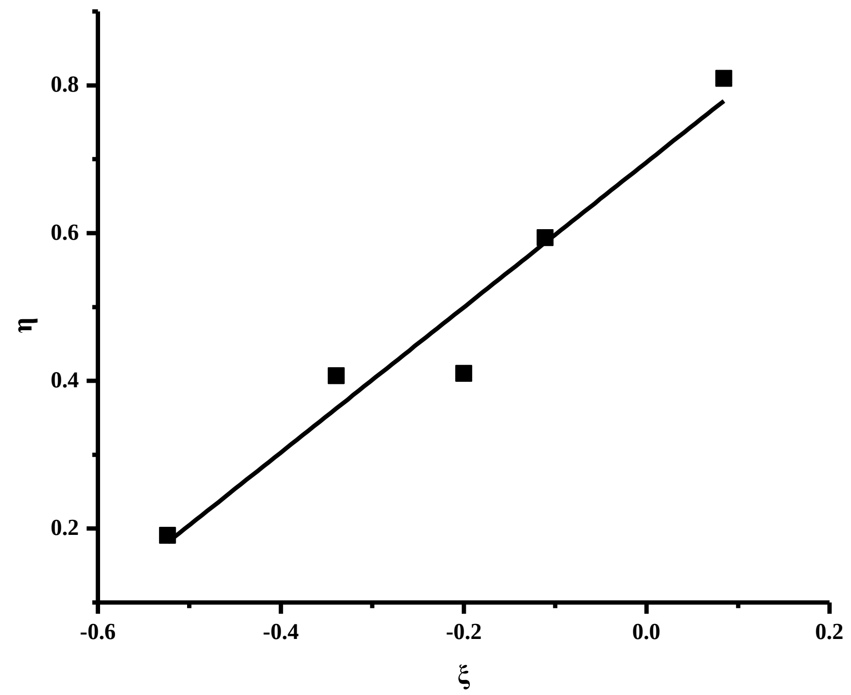

Figure S4. Extended Kelen- Tüdos plot for the PNVP-stat-PIBMA copolymers synthesized via free radical copolymerization.

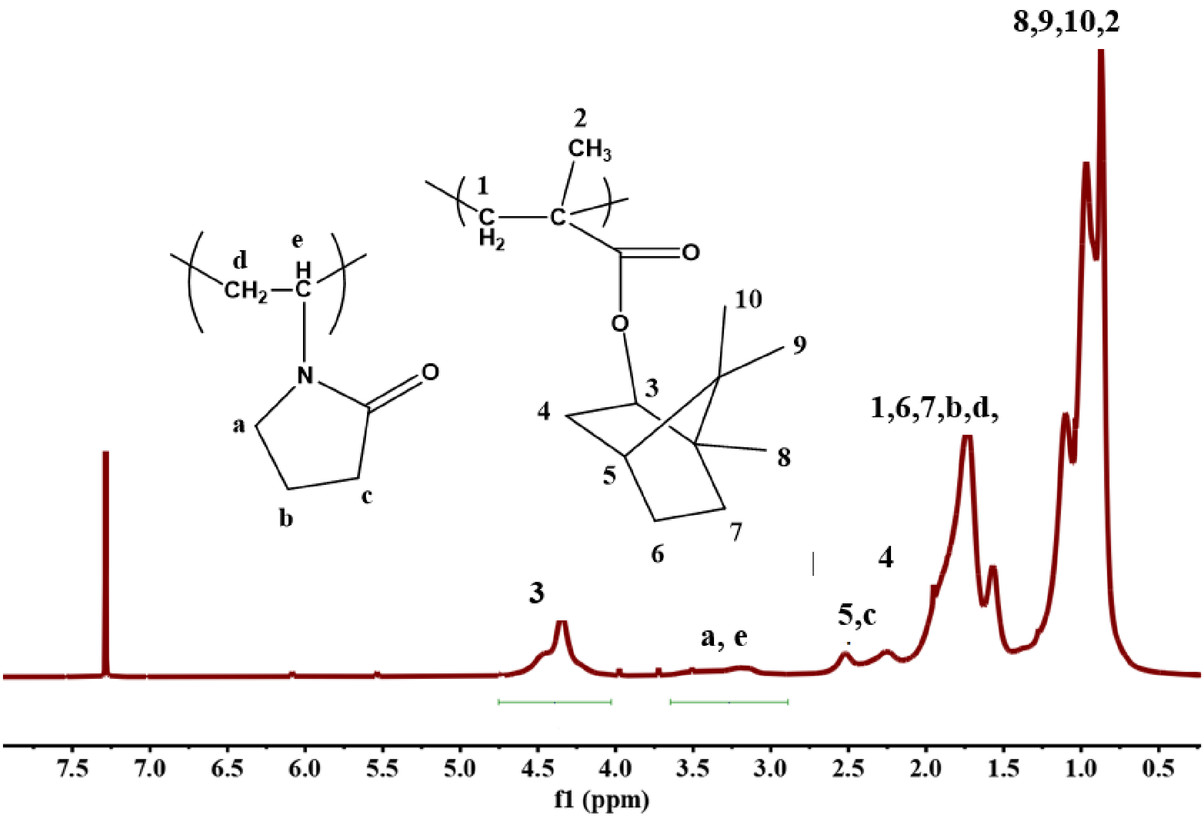

Figure S5. ^{1}H-NMR spectrum of the sample R60/40 in $CDCl_3$.

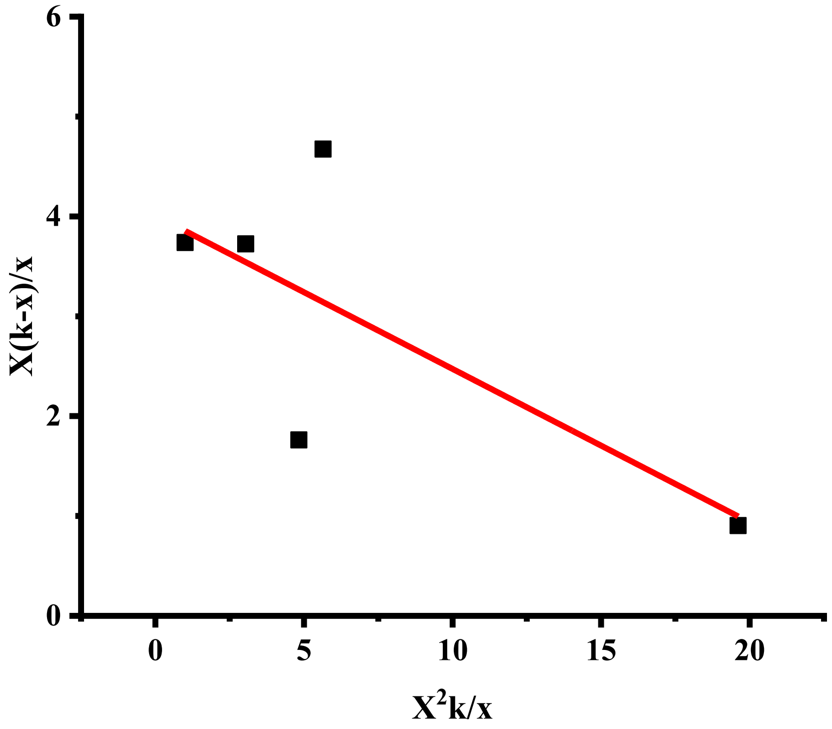

Figure S6. Barson-Fenn plot for the PNVP-stat-PIBMA copolymers synthesized via RAFT copolymerization.

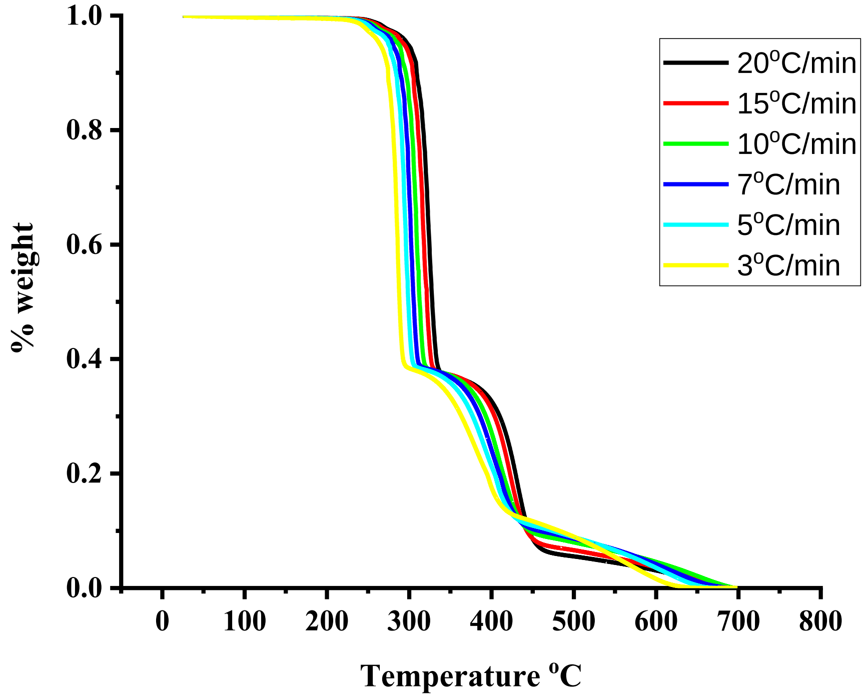

(**a**)

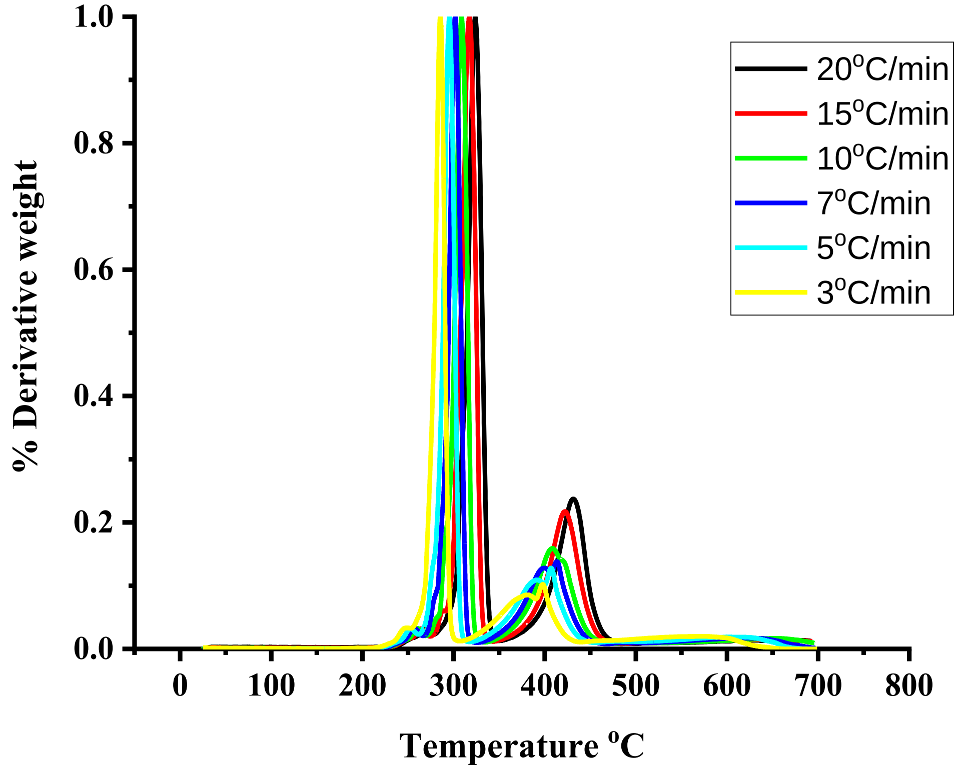

(**b**)

Figure S7. (**a**) TGA and (**b**) DTG thermograms for the sample R50/50 under different heating rates.

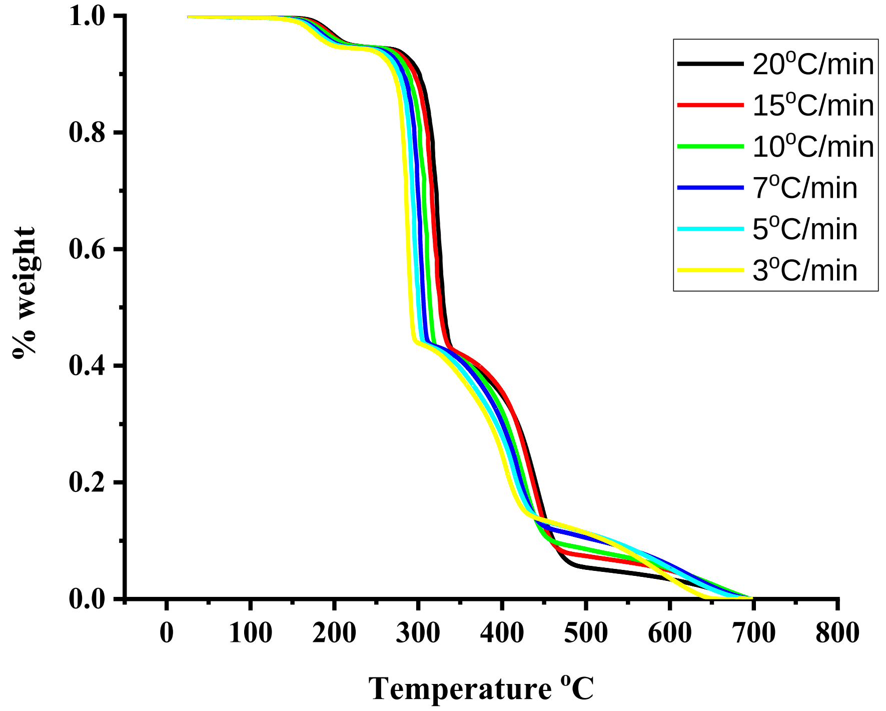

(**a**)

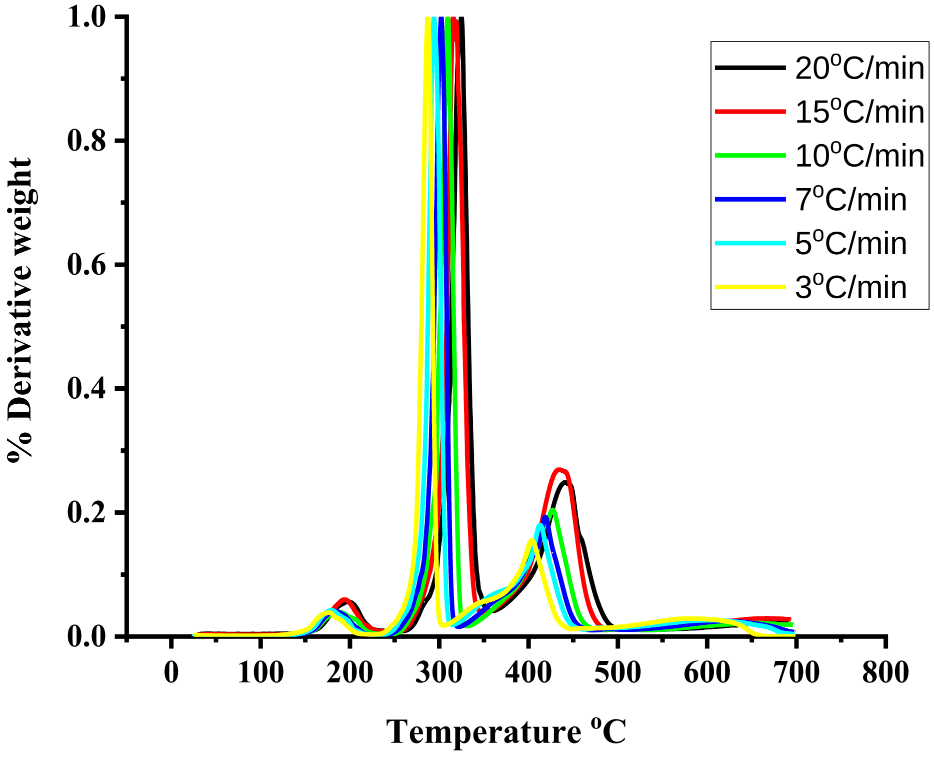

(**b**)

Figure S8. (**a**) TGA and (**b**) DTG thermograms for the sample R60/40 under different heating rates.

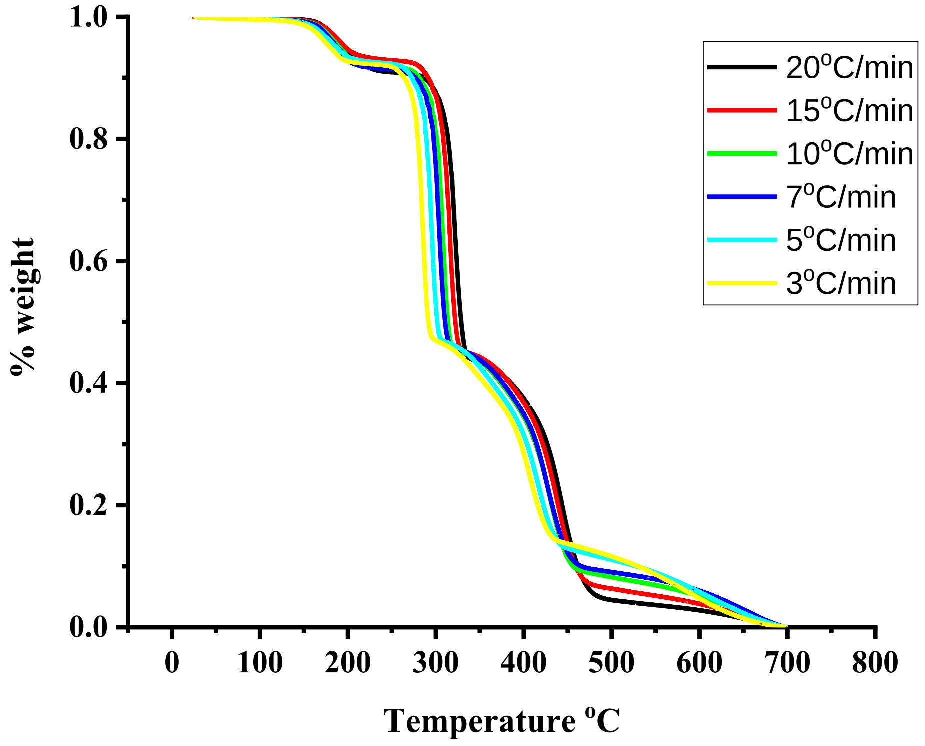

(**a**)

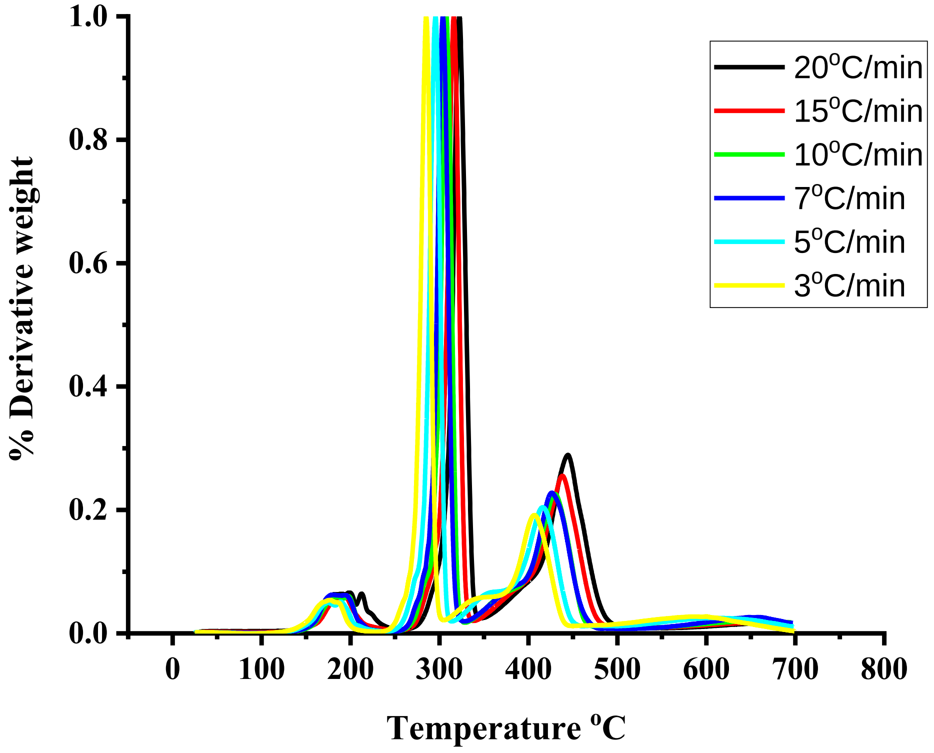

(**b**)

Figure S9. (**a**) TGA and (**b**) DTG thermograms for the sample R80/20 under different heating rates.

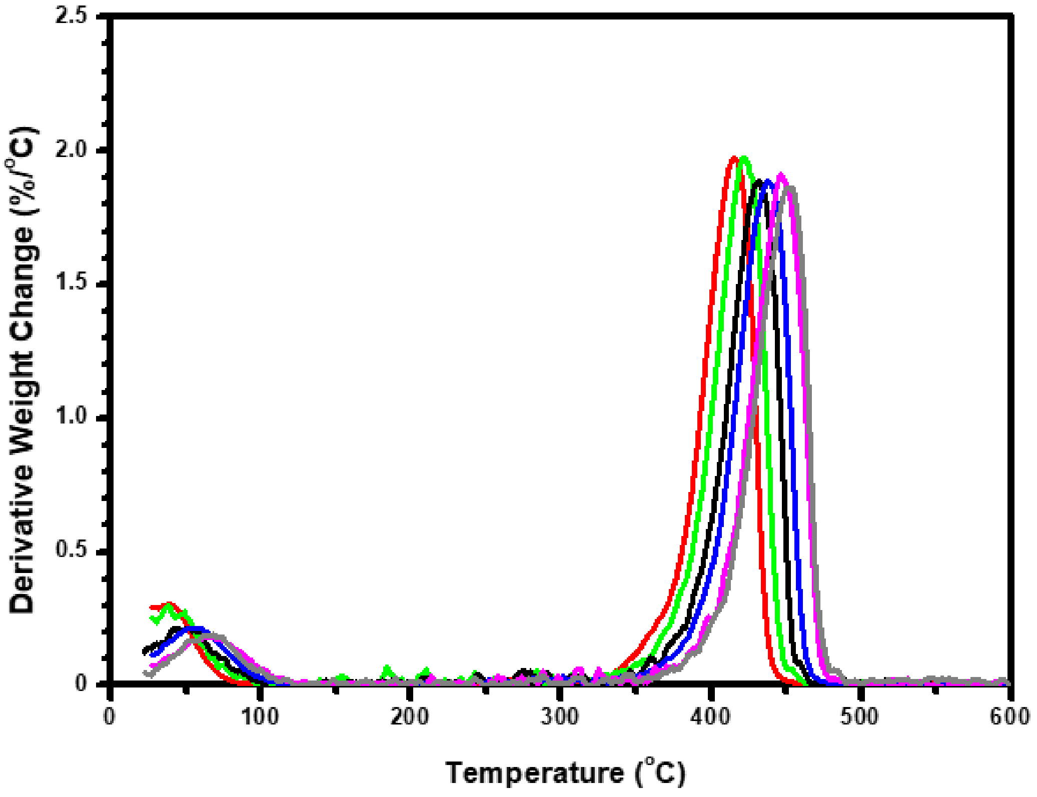

Figure S10. DTG thermograms (from left to right 3 to 20°C/min) for the PNVP homopolymer.

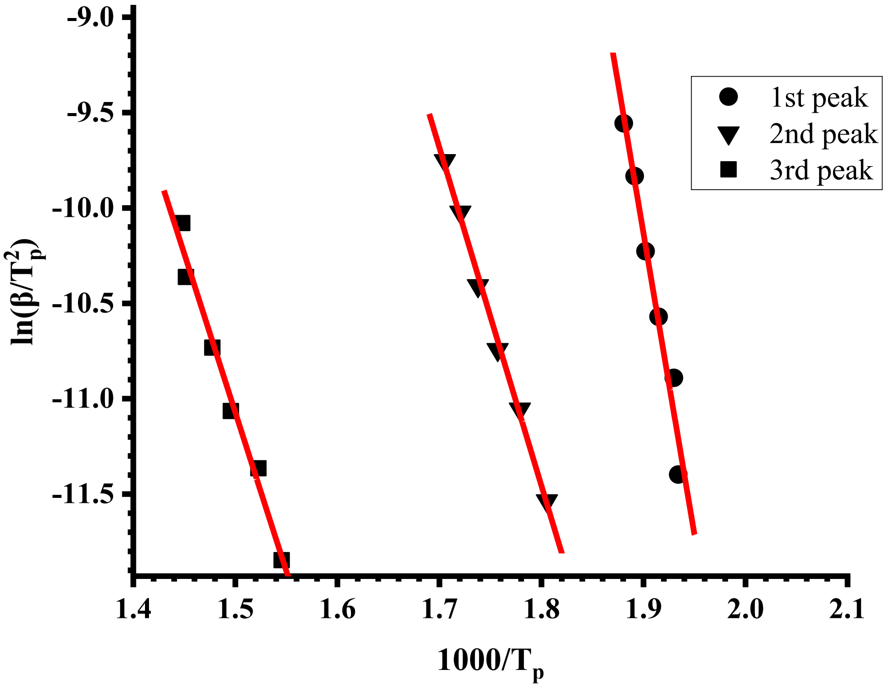

Figure S11. Kissinger plots for sample R20-80.

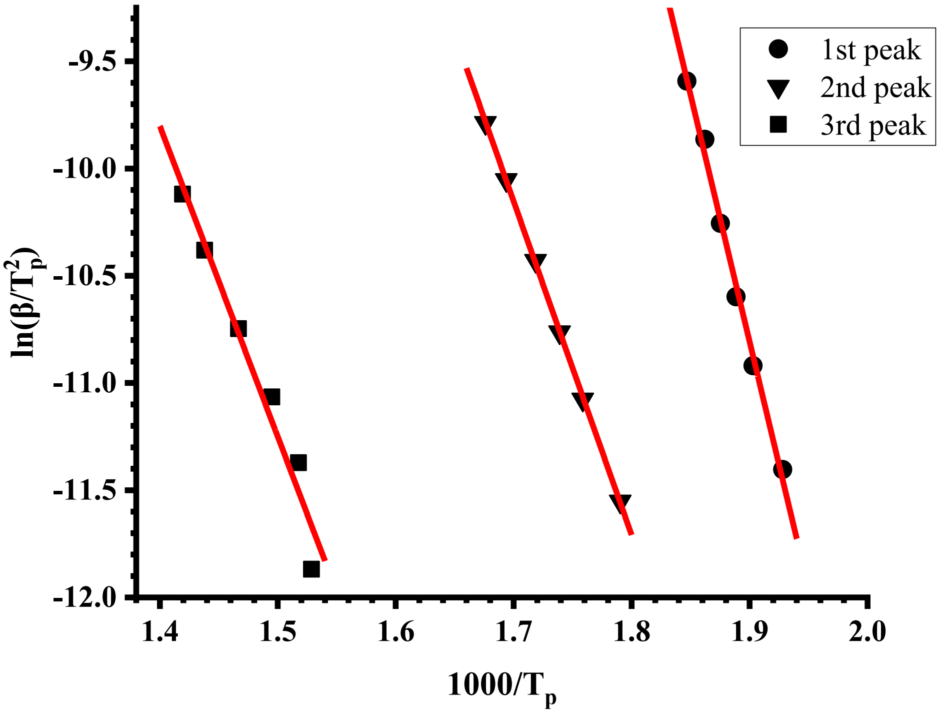

Figure S12. Kissinger plots for sample R50-50.

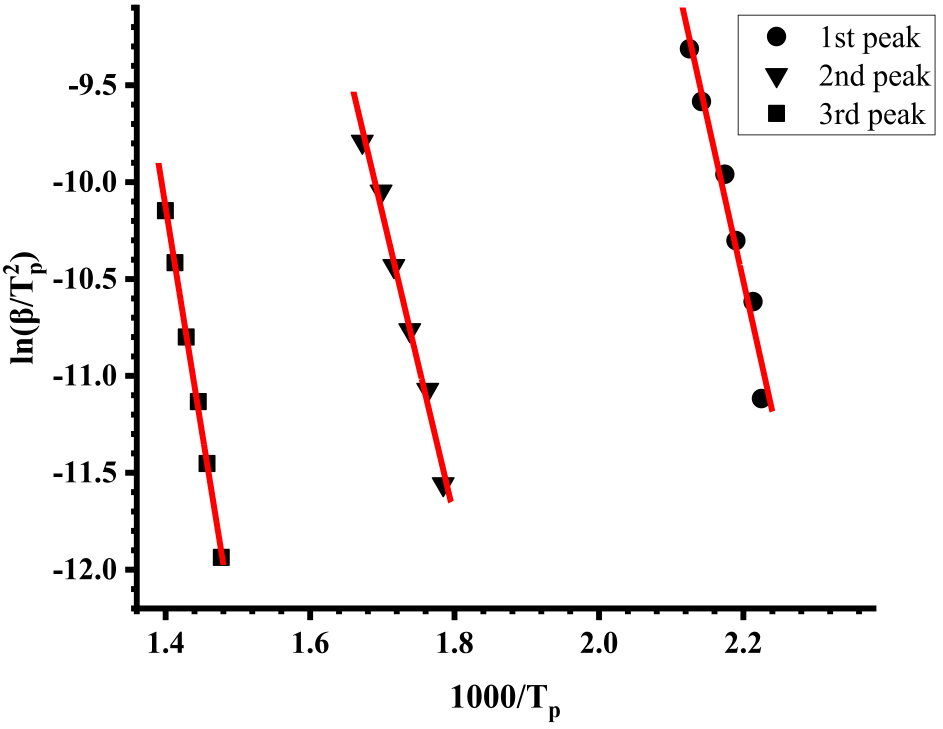

Figure S13. Kissinger plots for sample R60-40.

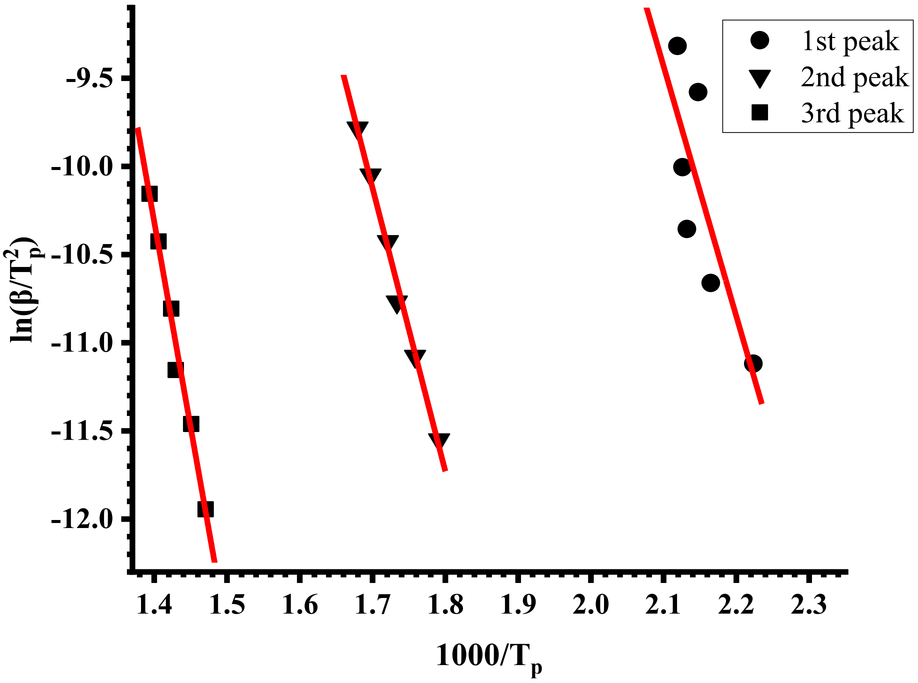

Figure S14. Kissinger plots for sample R80-20.

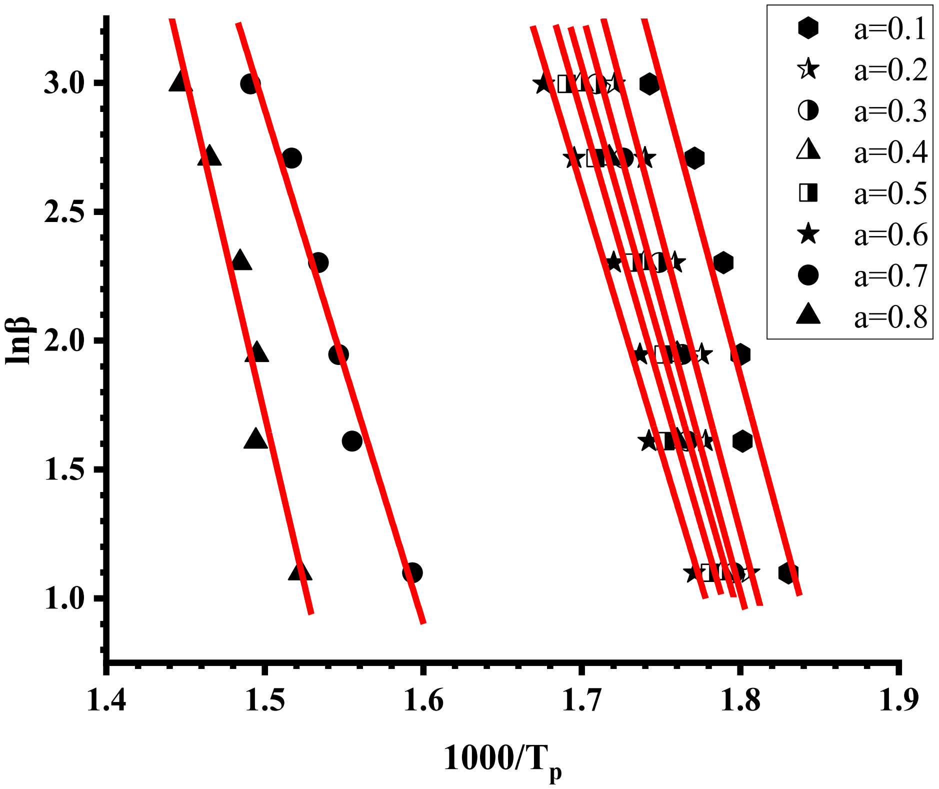

Figure S15. OFW plots for sample R40-60.

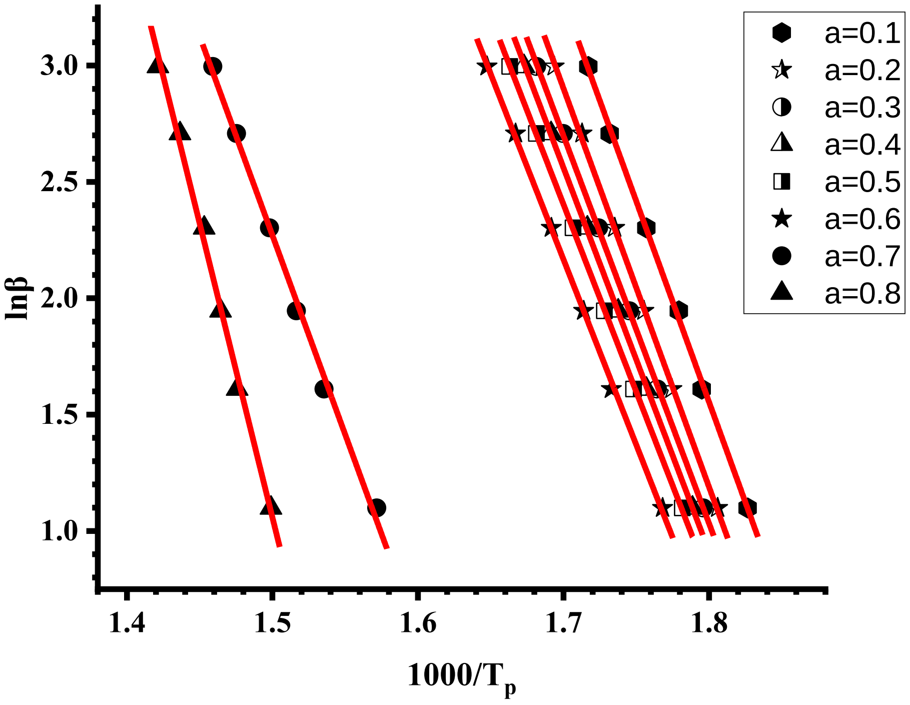

Figure S16. OFW plots for sample R50-50.

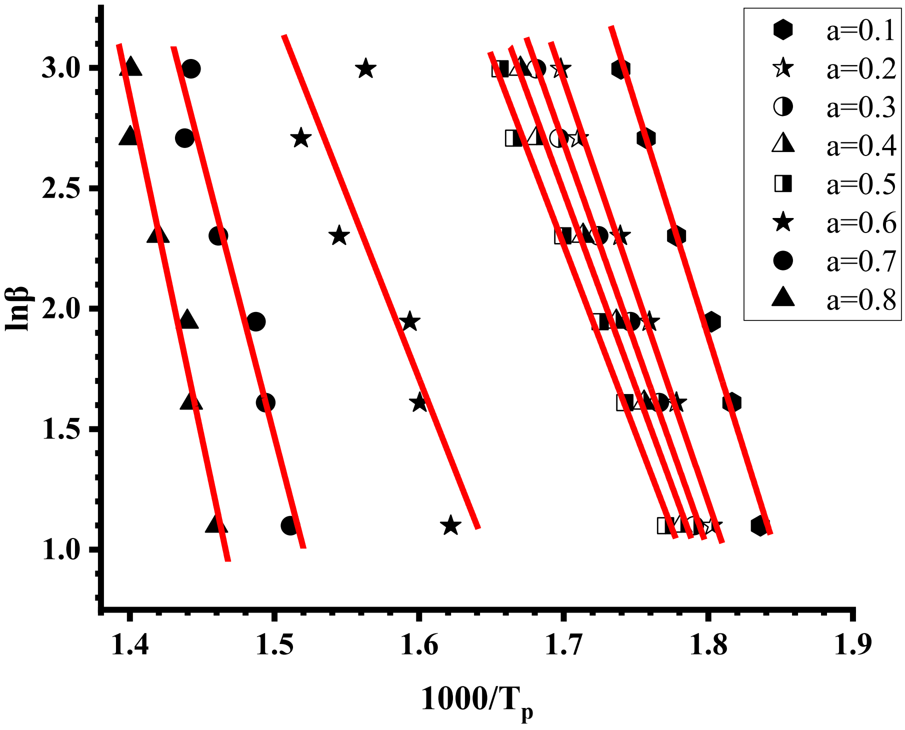

Figure S17. OFW plots for sample R60-40.

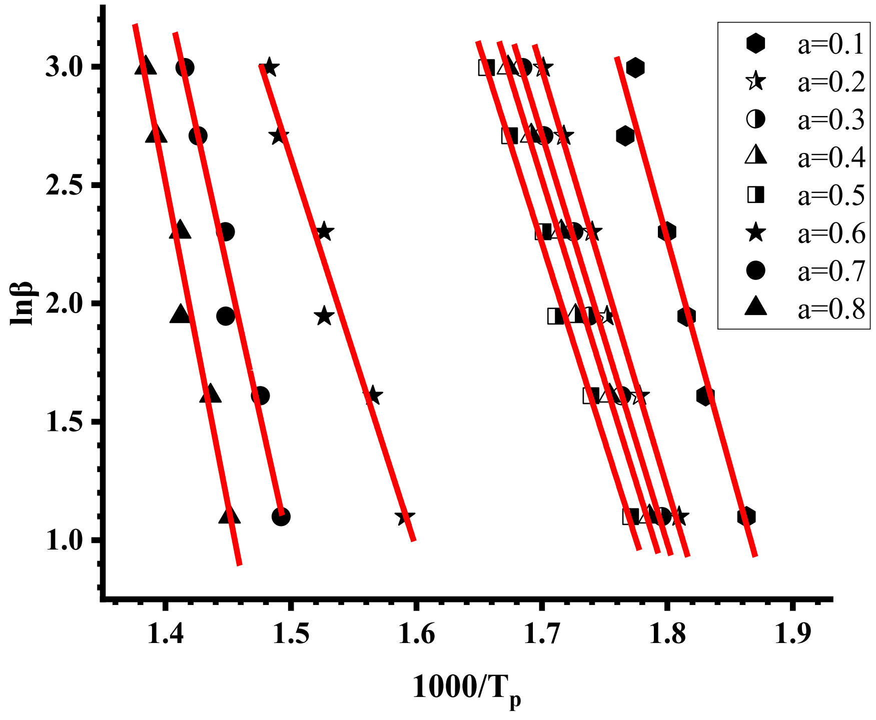

Figure S18. OFW plots for sample R80-20.

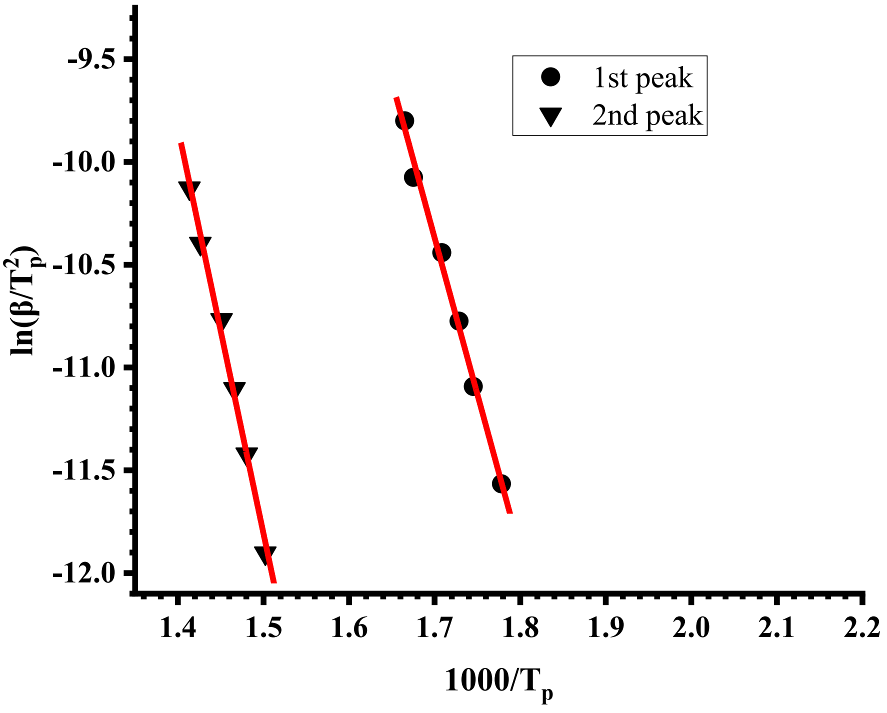

Figure S19. Kissinger plots for sample F50-50.

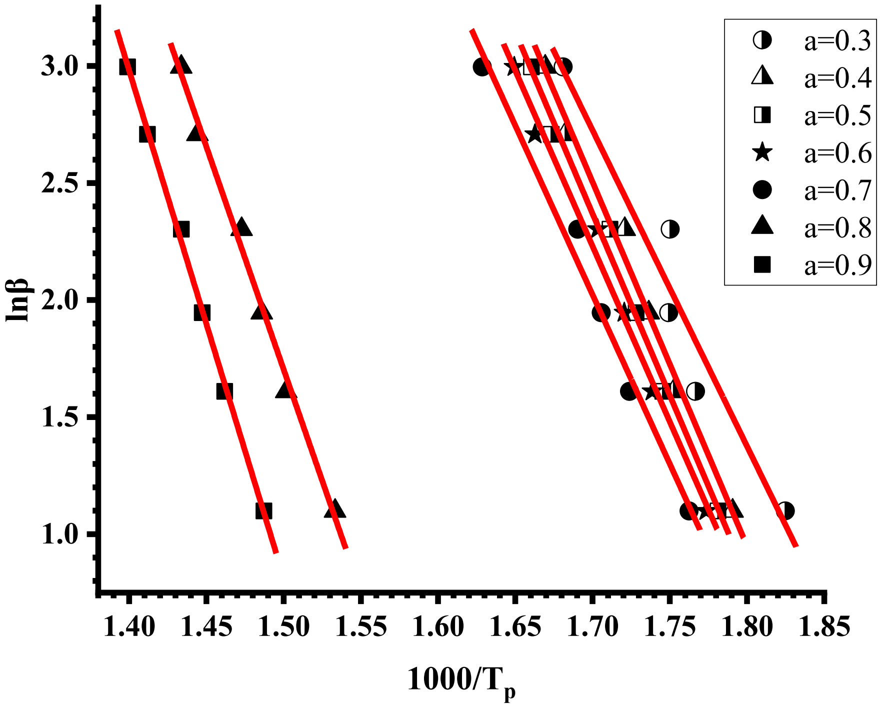

Figure S20. OFW plots for sample F50-50.

Table S1. Reagents quantities in free radical copolymerization.

Sample	mass of NVP, g	mass of IBMA, g	AIBN, g
F20/80	1.5284	11.3928	0.0135
F40/60	3.0148	8.5224	0.0124
F50/50	4.0312	7.5389	0.0119
F60/40	6.0054	7.5609	0.0143
F80/20	8.0016	3.7930	0.0125

Table S2. Reagents quantities in RAFT copolymerization[a].

Sample	mass of NVP, g	moles of NVP	mass of IBMA, g	moles of IBMA	CTA, g	moles CTA	AIBN, g	AIBN moles
R20/80	1.1170	0.0101	8.9008	0.0401	0.1060	0.000502	0.016	0.000100
R40/60	2.5625	0.0231	7.4402	0.0335	0.1200	0.000576	0.018	0.000113
R50/50	3.3462	0.0301	6.7075	0.0302	0.0128	0.000603	0.020	0.000121
R60/40	4.2127	0.0380	5.6662	0.0255	0.1350	0.000635	0.021	0.000127
R80/20	6.7589	0.0609	3.3793	0.0152	0.1610	0.000761	0.025	0.000161

[a] 50% w/v dioxane solutions.

Table S3. Reactivity Ratios of NVP and IBMA in RAFT copolymerization.

Method	r_{NVP}	r_{IBMA}
Fineman-Ross	-0.020	4.206
Inv. Fineman-Ross	-0.088	3.852
Kelen-Tüdos	-0.041	3.813

Table S4. DTG data for PNVP.

heat rate (β)	T_{onset} (K)	$T_{terminal}$ (K)	T_{peak} (K)
3	615.44	719.41	688.57
5	632.73	731.86	694.66
7	637.33	734.73	704.03
10	641.70	738.02	710.51
15	655.93	748.96	719.39
20	658.76	759.45	723.86

Table S5. DTG data for PIBMA.

heat rate (β)	T_{onset} 1st peak (°C)	$T_{terminal}$ 1st peak (°C)	T_{p1}(°C)
3	200.64	276.52	235.62
5	201.70	285.54	240.01
7	205.42	291.38	245.83
10	200.11	288.72	242.28
15	207.01	280.77	248.21
20	213.91	300.40	250.25
	**(continuation)**		
**heat rate (β)**	**T_{onset} 2nd peak (°C)**	**$T_{terminal}$ 2nd peak (°C)**	**T_{p2}(°C)**
3	276.52	306.24	285.55
5	285.54	319.50	295.22
7	291.38	327.46	302.12
10	288.72	328.02	301.32
15	280.77	337.54	309.40
20	300.40	342.32	314.69
	**(continuation)**		
**heat rate (β)**	**T_{onset} 3rd peak (°C)**	**$T_{terminal}$ 3rd peak (°C)**	**T_{p3}(°C)**
3	332.24	403.87	376.53
5	338.60	416.61	391.11
7	344.97	428.28	401.32
10	346.56	430.94	408.50
15	353.99	439.96	416.27
20	357.71	447.38	424.37

Table S6. DTG data for R20-80.

heat rate (β)	T_{onset} 1st peak (°C)	$T_{terminal}$ 1st peak (°C)	T_{p1}(°C)
3	218.68	256.89	243.38
5	226.11	257.42	245.03
7	227.70	264.85	249.08
10	231.42	270.15	252.60
15	234.07	273.34	255.55
20	240.22	273.57	258.57
	**(continuation)**		
**heat rate (β)**	**T_{onset} 2nd peak (°C)**	**$T_{terminal}$ 2nd peak (°C)**	**T_{p2}(°C)**
3	256.89	300.93	280.76
5	257.42	311.01	289.00
7	264.85	317.91	295.98
10	270.15	322.69	302.27
15	273.34	330.65	308.03
20	273.57	335.43	313.23
	**(continuation)**		
**heat rate (β)**	**T_{onset} 3rd peak (°C)**	**$T_{terminal}$ 3rd peak (°C)**	**T_{p3}(°C)**
3	316.85	415.02	373.92
5	320.03	430.94	383.60
7	331.71	434.65	395.46
10	340.20	442.61	403.70
15	351.34	449.51	415.70
20	356.65	455.50	417.48

Table S7. DTG data for R40-60.

**heat rate (β)**	**T_{onset} 1st peak (°C)**	**$T_{terminal}$ 1st peak (°C)**	**T_{p1}(°C)**
3	218.68	253.70	241.84
5	223.99	262.19	247.16
7	225.58	260.07	245.54
10	228.23	264.32	248.98
15	233.01	265.91	250.01
20	235.66	270.68	257.09
	**(continuation)**		
**heat rate (β)**	**T_{onset} 2nd peak (°C)**	**$T_{terminal}$ 2nd peak (°C)**	**T_{p2}(°C)**
3	253.70	303.58	285.47
5	262.19	313.66	294.88
7	260.07	315.26	294.07
10	264.32	323.22	300.69
15	265.91	333.83	308.76
20	270.68	339.14	313.76
	**(continuation)**		
**heat rate (β)**	**T_{onset} 3rd peak (°C)**	**$T_{terminal}$ 3rd peak (°C)**	**T_{p3}(°C)**
3	315.26	425.10	372.93
5	323.75	440.49	388.12
7	327.99	442.08	399.82
10	332.24	447.92	399.76
15	342.32	461.71	405.51
20	352.93	463.30	421.18

Table S8. DTG data for R50-50.

heat rate (β)	T_{onset} 1st peak (°C)	$T_{terminal}$ 1st peak (°C)	T_{p1}(°C)
3	218.15	253.70	245.48
5	221.86	262.72	252.36
7	224.52	267.50	256.37
10	230.36	270.68	260.10
15	235.13	274.40	263.88
20	240.97	280.23	268.32
	**(continuation)**		
**heat rate (β)**	**T_{onset} 2nd peak (°C)**	**$T_{terminal}$ 2nd peak (°C)**	**T_{p2}(°C)**
3	253.70	303.05	285.41
5	262.72	315.79	295.50
7	267.50	322.15	301.91
10	270.68	330.11	308.74
15	274.40	337.14	317.27
20	280.23	347.63	323.48
	**(continuation)**		
**heat rate (β)**	**T_{onset} 3rd peak (°C)**	**$T_{terminal}$ 3rd peak (°C)**	**T_{p3}(°C)**
3	312.60	434.12	381.04
5	323.22	450.57	385.78
7	331.71	453.75	395.75
10	339.14	461.18	408.64
15	349.75	470.73	422.20
20	353.99	478.16	431.42

Table S9. DTG data for R60-40.

heat rate (β)	T_{onset} 1st peak (°C)	$T_{terminal}$ 1st peak (°C)	T_{p1}(°C)
3	125.21	207.89	176.28
5	119.00	217.21	178.63
7	123.33	230.89	183.56
10	142.62	233.37	186.76
15	148.83	229.02	193.72
20	151.94	232.75	197.38
	**(continuation)**		
**heat rate (β)**	**T_{onset} 2nd peak (°C)**	**$T_{terminal}$ 2nd peak (°C)**	**T_{p2}(°C)**
3	236.48	304.23	287.24
5	240.83	314.50	294.05
7	238.34	322.26	302.18
10	247.67	330.34	309.62
15	258.86	348.37	315.67
20	263.83	358.31	324.85
	**(continuation)**		
**heat rate (β)**	**T_{onset} 3rd peak (°C)**	**$T_{terminal}$ 3rd peak (°C)**	**T_{p3}(°C)**
3	307.34	447.82	403.8
5	316.67	456.53	412.98
7	323.50	467.72	418.74
10	335.31	472.69	426.89
15	350.85	486.36	434.54
20	362.67	500.66	441.32

Table S10. DTG data for R80-20.

heat rate (β)	T_{onset} 1st peak (°C)	$T_{terminal}$ 1st peak (°C)	T_{p1}(°C)
3	114.68	222.40	176.61
5	120.53	236.72	188.77
7	126.88	235.66	195.86
10	129.53	238.31	197.20
15	135.37	244.68	192.53
20	138.56	247.87	198.75
	**(continuation)**		
**heat rate (β)**	**T_{onset} 2nd peak (°C)**	**$T_{terminal}$ 2nd peak (°C)**	**T_{p2}(°C)**
3	234.60	303.58	285.03
5	242.03	312.60	295.51
7	251.58	326.93	303.7
10	253.70	327.99	307.83
15	258.48	337.54	316.02
20	263.25	344.97	322.30
	**(continuation)**		
**heat rate (β)**	**T_{onset} 3rd peak (°C)**	**$T_{terminal}$ 3rd peak (°C)**	**T_{p3}(°C)**
3	308.89	446.32	406.80
5	242.03	456.94	416.08
7	329.05	479.75	426.31
10	253.70	481.88	429.41
15	258.48	491.96	438.12
20	263.25	506.82	444.36

Table S11. Activation energies Ea (KJ/mol) by the Kissinger method.

sample	slope1	slope2	Ea1	Ea2
**F50-50**	-15.22	-19.71	126.54	163.87

Table S12. Activation energies Ea (KJ/mol) by the OFW method.

conversion	F50-50
a=0.1	22.45
a=0.2	39.91
a=0.3	112.24
a=0.4	129.37
a=0.5	129.03
a=0.6	124.71
a=0.7	120.14
a=0.8	158.22
a=0.9	180.08
